# In silico drug repositioning using deep learning and comprehensive similarity measures

**DOI:** 10.1186/s12859-020-03882-y

**Published:** 2021-06-01

**Authors:** Hai-Cheng Yi, Zhu-Hong You, Lei Wang, Xiao-Rui Su, Xi Zhou, Tong-Hai Jiang

**Affiliations:** 1 The Xinjiang Technical Institute of Physics and Chemistry, Chinese Academy of Sciences, Urumqi, 830011 China; 2 University of Chinese Academy of Sciences, Beijing, 100049 China

**Keywords:** Drug repositioning, Drug–disease interaction, Gated recurrent units, Gaussian interaction profile kernel, Machine learning

## Abstract

**Background:**

Drug repositioning, meanings finding new uses for existing drugs, which can accelerate the processing of new drugs research and development. Various computational methods have been presented to predict novel drug–disease associations for drug repositioning based on similarity measures among drugs and diseases. However, there are some known associations between drugs and diseases that previous studies not utilized.

**Methods:**

In this work, we develop a deep gated recurrent units model to predict potential drug–disease interactions using comprehensive similarity measures and Gaussian interaction profile kernel. More specifically, the similarity measure is used to exploit discriminative feature for drugs based on their chemical fingerprints. Meanwhile, the Gaussian interactions profile kernel is employed to obtain efficient feature of diseases based on known disease-disease associations. Then, a deep gated recurrent units model is developed to predict potential drug–disease interactions.

**Results:**

The performance of the proposed model is evaluated on two benchmark datasets under tenfold cross-validation. And to further verify the predictive ability, case studies for predicting new potential indications of drugs were carried out.

**Conclusion:**

The experimental results proved the proposed model is a useful tool for predicting new indications for drugs or new treatments for diseases, and can accelerate drug repositioning and related drug research and discovery.

## Background

Although the impressive advances have been witnessed in life sciences and technology and genomics over the past years. To bring a new drug to patients still takes ~ 15 years and 800 million to one billion of dollars [1–3]. Traditional drug research and development (R&D) process requires testing for side efforts and safety through cellular model systems, extensive animal model and clinical trial experimental validation. The average cost of new drug discovery has significantly increased and more than 90% of drug candidates fail during development, which caused pharmaceutical R&D tremendously expensive, time costing and high risky [3, 4]. This further directly led to a small quantity and high price of new drugs on the market. Drug repositioning or drug repurposing, identifying new clinical indications for those approved drugs has been used as an important strategy to maximize the potential usage of the existing drugs and increase the number of new drugs [5, 6]. Compared with the traditional drug R&D process, drug repositioning has two major advantages. The first is the safety of the approved drugs has been verified by rigorous clinical trials, the repositioning candidates have passed all necessary tests usual to de novo drug R&D, so these drugs are safe to use. Another advantage is drug repositioning has an abridged process of drug discovery and preparation, which means saving time and money.

In recent years, the establishment of online public databases on pharmacochemical properties, drug molecules chemical structure, drug–drug interactions, disease–disease interactions, related genomic sequences and side efforts has promoted the study of drug–disease interactions and drug repositioning [7]. Such as KEGG [8], OMIM [9], CMap [10], DrugBank [11], STITCH [12] and ChEMBL [13]. The goal of drug repositioning is to find potential indications for existing approved drugs and apply the new identified drug candidates to the clinical treatment for other disease than originally targeted disease. Integrated data from these various sources, to date, many machine learning methods are developed [14–25].

For instance, Chiang et al. conducted a ‘guilt-by-association’ network-based model to predict potential drug–disease associations, this method assumes that if the two diseases have similar treatment profiles, then the drug used for only one of the two diseases can be used for the other, thus recommending the new use of a drug. However, this approach tends to older drugs with multiple different uses and diseases with manifold different treatments [26]. Gottlieb et al. [27] demonstrated a method for large-scale prediction of drug indications, named PREDICT, which uses comprehensive drug–drug and disease–disease similarity measures to obtain discriminative features. Napolitano et al. [28] proposed a multi-class Support Vector Machine (SVM) classifier to predict novel drug–disease interactions and they defined drug similarities by using combined drug datasets. Moreover, some network-based methods also be put forward in recent years [29, 30]. Wu et al. [31] introduced a weighted drug–disease heterogeneous network to predict new use of drug by clustering based on experimental proved drug–target interactions and gene–disease relationships. Wang et al. [32] also constructed a heterogeneous network integrated drug targets, diseases and drugs into a unified framework, which can rank candidate drugs for each disease by an iterative approach. Martinez et al. [33] proposed DrugNet to perform drug–disease and disease–drug prioritization based on a network-based prioritization method, which can integrate extensive types of data from complex networks involving interconnected drugs, proteins and diseases.

More recently, some recommendation system based methods have been developed for computational drug discovery [34, 35]. Luo et al. [5] presented MBiRW model to identify new interactions for known drugs, which applied comprehensive similarity measures and Bi-Random walk algorithm. Thereafter, Nagaraj et al. [4] developed a novel drug discovery strategy DrugPredict, which combined computational model with biological testing in cell line in order to rapidly identify novel drug candidates for epithelial ovarian cancer. Their work exploited unique repositioning opportunities rendered by a vast amount of disease genomics, phenomics, treatments and genetic pathway [4]. Matrix factorization methods have also been used to identify novel drug–disease interactions, which takes one input matrix and obtained two related matrices as output, while the two are multiplied to approximate the originally input matrix, e.g. kernel Bayesian matrix factorization, collaborative matrix factorization method and so on. Most existing methods rely on the properties of some important drugs or diseases to exploit the drug similarity and disease similarity measures. However, there are some known interactions between drugs and diseases that previous studies have not considered to utilize, which yet have valuable information can be exploited to improve similarity measures.

**Fig. 1:**
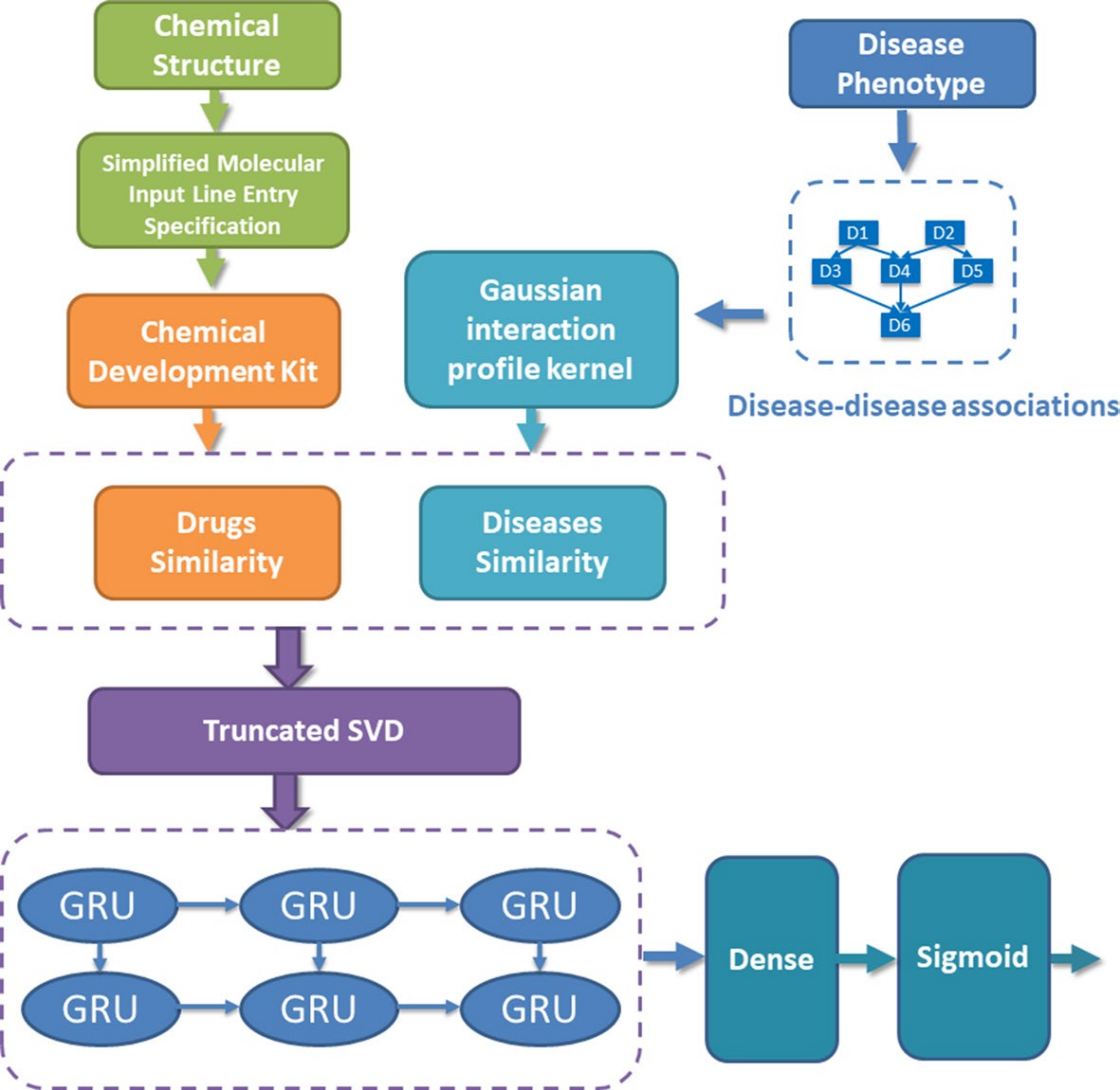
The workflow of DDIPred

In this study, we propose a deep learning model for potential Drug–Disease Interactions Prediction, named DDIPred. It applied gated recurrent neural network for predicting new indications of existing drugs using comprehensive similarity measures and Gaussian interaction profile kernel features. The workflow of this study is demonstrated as shown in Fig. 1. More specifically, the similarity measures are calculated based on drug chemical structures, disease phenotypes and known drug–disease interactions. Furthermore, the Gaussian interaction profile (GIP) kernel was applied to exploit effective feature of drug and disease based on known drug–disease interactions. The truncated singular value decomposition (TSVD) is further used to reduce the dimensionality of these combined two feature [17]. Finally, we fed these discriminative features into deep gated recurrent units (GRU) model as input to learn and predict the novel drug–disease interactions, which means potential new use of existing drugs. Moreover, the performance of the proposed model is evaluated on two gold standard datasets under ten-fold cross-validation. And we further made case studies to verify the predictive ability of our model. Experimental results demonstrate that the proposed model has the superior capability to discover potential new use of drugs.

## Materials and methodology

In this section, the dataset used in this study will be introduced first. And then, based on the basic hypothesis that the similar drugs have similar indications, we proposed a novel deep learning approach of integrating comprehensive similarity measures and Gaussian interaction profile kernel with GRU model to predict potential drug–disease interactions. We will present the details of similarity measures and Gaussian interaction profile kernel and the implement of GRU model. Meanwhile, we will also describe the comparison models, experimental methods, and the evaluation criteria in this section.

### Benchmark datasets

To evaluate the performance of our model, we selected two widely used benchmark datasets including Fdataset and Cdataset. The gold standard dataset Fdataset is obtained from Gottlieb et al.’s work [27], which is made up of multiple data sources. More concretely, for this dataset, there are 1933 known associations between drugs and diseases and 593 drugs from DrugBank [36] and 313 diseases registered in OMIM [9] (the Online Mendelian Inheritance in Man). We also carried out another benchmark dataset Cdataset at the same time, this dataset is firstly presented in Luo et al.’s paper [5]. There are 2532 drug–disease associations in this dataset, including 409 diseases and 663 drugs. Each dataset consists of three matrices: drug–drug similarity matrix $S_{D} \in R^{m \times m}$, disease-disease similarity matrix $S_{d} \in R^{n \times n}$ and drug–disease interactions matrix $I \in R^{m \times n}$. $S_{D}$ and $S_{d}$ are symmetric matrices and each row or column element represents the similarity between a drug and other drugs, a disease and other diseases, respectively. The details of similarity calculation is given in next section. The *m* rows of matrix $I$ indicate *m* drugs, *n* columns represent *n* diseases, when drug $D_{i}$ have association with disease $d_{j}$, set the element $I\left( {i,j} \right)$ to 1, else set to 0. The interacting drug–disease pairs are used as positive samples, and the same number of pairs without known interaction are randomly selected as negative samples. The details of these two datasets are shown in Table 1.

**Table 1:** The details of the two drug–disease associations benchmark datasets

Dataset	Number of drugs	Number of diseases	Interaction pairs
Fdataset	593	313	1933
Cdataset	663	409	2532

### Similarity measures

Follow the description above, the drugs similarity is calculated based on the chemical structure information, which comes from drug-related properties [5]. More concretely, the similarity between two drugs is calculated by the Chemical Development Kit [37] of their 2D chemical fingerprints, which use the Simplified Molecular Input Line Entry Specification (SMILES) [38] of all drugs that downloaded from DrugBank. Moreover, the correlation between two drugs’ similarity and their common diseases are analyzed and set those similarity that is not discriminative close to 0. The similarity are adjusted using the logistic regression function which has been used to modify the diseases-genes associations similarity by [39]. The function can be defined as follow:

1 $$L\left( {\text{x}} \right) = \frac{1}{{1 + e^{{\left( {ax + b} \right)}} }}$$

where *x* represents the similarity value, *a* and *b* are adjusting parameters. And then, the drugs are clustered based on known drug–disease associations by using a graph clustering method, ClusterONE [40], which has been employed to detect valuable modules for drug repositioning [5, 31, 41]. The cohesiveness of a cluster M could be defined by ClusterONE as follows:

2 $$f\left( M \right) = \frac{{C_{in} \left( M \right)}}{{(C_{in} \left( M \right) + C_{bound} \left( M \right) + P\left( M \right))}}$$

where $C_{in} \left( M \right)$ indicates the total weight of edges within a set of vertices *M*, $C_{bound} \left( M \right)$ stands for the total weight of edges connecting this set to the remaining of group, and *P*(*M*) is the penalty term [5].

### Gaussian interaction profile kernel

For diseases, we adopted Gaussian interaction profile kernel [42] to obtain the representation of disease–disease associations [43]. Based on the assumption that the diseases with a similar interaction pattern with drugs are likely to show similar interaction behavior with new drugs [42]. Similar assumptions can also be applied to drugs. Suppose ($D_{i}$, $D_{j}$) indicates two different drugs, while ($d_{i}$, $d_{j}$) represents two different diseases. Their gaussian interaction profile kernel similarity *KG* can calculation as follows:

3 $$KG_{disease} \left( {d_{i} , d_{j} } \right) = {\text{exp}}\left( { - \alpha_{d} \left\| d_{i} - d_{j}^{2}\right\| } \right)$$

4 $$\alpha_{d} = \frac{{{\alpha_{d}}^{{\prime}} }}{{\left( {\frac{1}{nd}\mathop \sum \nolimits_{i = 1}^{{n_{d} }} \left| {y_{{d_{i} }} } \right|^{2} } \right)}}$$

Here, for simplicity, the ${\alpha_{d}}^{{\prime}}$ is set to 0.5, and the $n_{d}$ stands for the number of the diseases, which is inspired by [42]. Then, the matrix decomposition algorithm TSVD was further applied to reduce the dimension of these features.

### Implementation of gated recurrent units neural network

In order to overcome several known defects of standard Recurrent Neural network (RNN) model, a series of improved models has been proposed in deep learning field. Among them, the Long short term memory (LSTM) [44, 45] and other similar variant models have the best performance and are widely used in a many fields [46–48]. The main reason for their effectiveness is the pull-in of gated mechanisms. The Gated Recurrent Units (GRU) was proposed by Cho et al. [49], which has only resetting gate and updating gate and all memory contents are fully open to each timestep. We follow the similar calculation process in [50].

The update gate $u_{t}$ is calculated by:

5 $$z_{t} = sigmoid\left( {W_{z} i_{t} + U_{t} h_{t - 1} - b_{z} } \right)$$

here, the $i_{t}$ indicates the input vector of GRU, $h_{t - 1}$ stands for the previous output of model, $W_{z}$, $U_{z}$ and $b_{z}$ are forward, recurrent matrices and biases for update gate, respectively. Similar to the process of update gate, the computed process of reset gate can be defined as follows:

6 $$r_{t} = sigmoid\left( {W_{r} i_{t} + U_{r} h_{t - 1} - b_{r} } \right)$$

where the parameters are same as above. Moreover, the candidate memory state $c_{t}$ can be computed by:

7 $$c_{t} = \sigma \left( {W_{h} i_{t} + U_{h} \left( {r_{t} *h_{t - 1} } \right) - b_{h} } \right)$$

where $\sigma_{h}$ is the tanh function and $*$ means an element-wise multiplication. Finally, the memory state $h_{t}$ of the GRU model is defined as:

8 $$h_{t} = \left( {1 - z_{t} } \right)h_{t - 1} + z_{t} c_{t}$$

In practice, the GRU model is implemented based on Keras framework [51]. Considering the limited scale of the problem, we set the number of hidden neurons in the GRU input layer to 128 and add a Dense layer (fully connected layer) behind the output layer as the classifier to reduce the final prediction probability results. The sigmoid function is employed as activation function, its mathematical behaviors can be expressed as follows:

9 $$\upsigma = {\text{sigmoid}}\left( x \right) = \frac{1}{{\left( {1 + e^{ - x} } \right)}}$$

before activation layer, we applied Dropout to reduce overfitting and enhance the model’s robustness [52]. The parameter of dropout was set to 0.25. And the binary cross-entropy was used as loss function, which corresponding to sigmoid activation function. Furthermore, loss function has significant influence to the performance of machine learning model. The binary cross-entropy can be defined as:

10 $$L\left( {{\text{t}},{\text{p}}} \right) = - \left( {\left( {1 - {\text{p}}} \right) \times \log \left( {1 - {\text{p}}} \right) + {\text{t}} \times {\text{log}}\left( {\text{p}} \right)} \right)$$

where *p* and *t* denote the prediction output and true label value. Moreover, we used the Adam optimizer the update the weights of model. The Adam integrated the advantages of both RMSProp and AdaGrad, which is popluar in this field [53].

### Performance evaluation metrics

In order to comprehensively evaluate the performance of our model, we follow the widely used evaluation indicators and strategies [54, 55]. The tenfold cross-validation was applied to evaluate the performance of DDIPred. In each validation, all data randomly divides into ten equal parts. Nine-fold data are taken as train data, the rest one-fold is taken as test data. To guarantee the unbiased comparison, it confirmed that there is no overlap between train data and test data. The final validation result is the mean value of tenfold with standard deviations. We follow the extensive used evaluation criteria, including accuracy (Acc), true positive rate (TPR), true negative rate (TNR), positive predictive value (PPV) and Matthews Correlation Coefficient (MCC) defined as:

11 $${\text{Acc}} = \frac{TN + TP}{{TN + TP + FN + FP}}$$

12 $${\text{TPR}} = \frac{TP}{{TP + FN}}$$

13 $${\text{TNR }} = \frac{TN}{{TN + FP}}$$

14 $${\text{PPV}} = \frac{TP}{{TP + FP}}$$

15 $${\text{MCC}} = \frac{TP \times TN - FP \times FN}{{\sqrt {\left( {TP + FP} \right)\left( {TP + FN} \right)\left( {TN + FP} \right)\left( {TN + FN} \right)} }}$$

where *TN* stands for the true negative number, *TP* represents the true positive number, *FN* denotes the false negative number and *FP* indicates the false positive number. Certainly, the Receiver Operating Characteristic (ROC) curve and the area under the ROC curve (AUC) are also adopted to evaluate the performance. And considering the specificity of the research task, the predicted top-N ranked results are more valuable for related drug development or disease treatment research. We also test the performance of model based on the count of accurately retrieved true drug–disease interactions.

## Results and discussion

In this study, we propose a deep learning model to predict potential drug–disease interactions, which can advance the discovery of new use of existing drugs or new treatment of diseases. In this section, we will systematically evaluate the performance of the model. Firstly, we evaluated the prediction capability of DDIPred on two benchmark datasets. And then, we compared it with other state-of-the-art models under the same experimental conditions. Furthermore, we made case studies to verify the practicability of the proposed method.

### Drug–disease interactions prediction capability evaluation

**Fig. 2:**
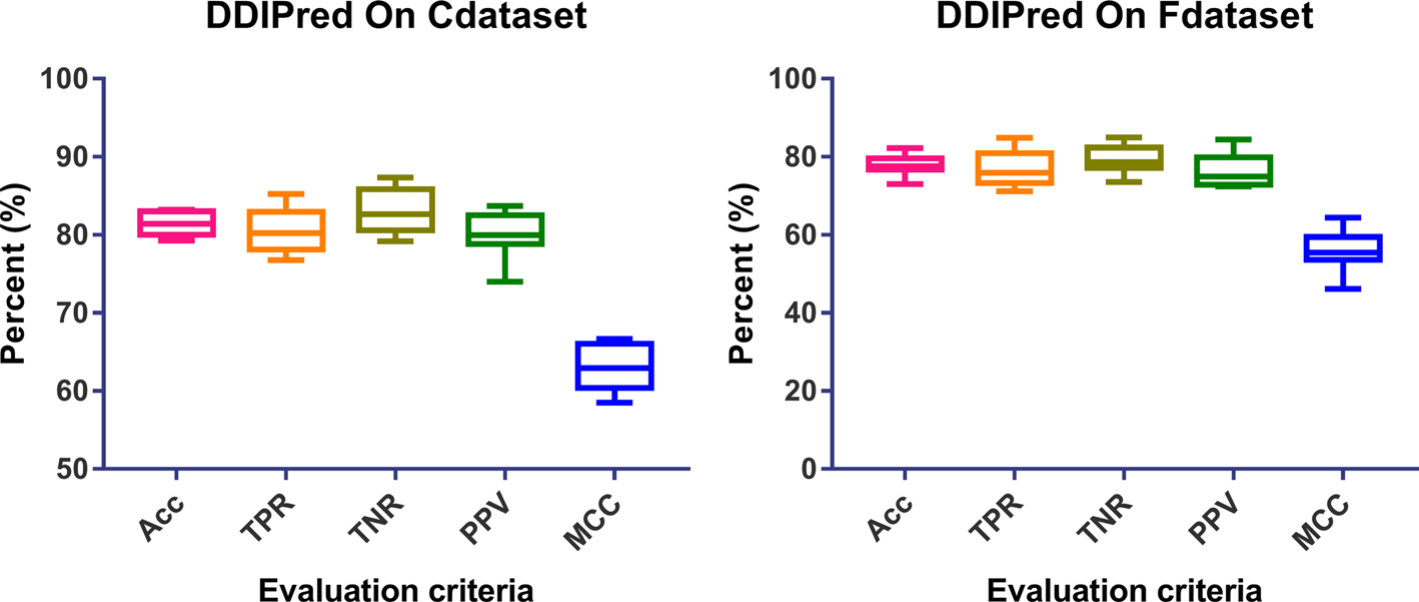
The performance of DDIPred on two benchmark datasets

First, the drug–disease interactions prediction capability of DDIPred is evaluated on two benchmark datasets Fdataset and Cdataset. The details of tenfold cross validation are listed at Tables 2 and 3 for Cdataset and Fdataset. The average values of tenfold cross-validation are taken as final report results as shown in Fig. 2.

**Table 2:** The tenfold cross-validation details on Cdataset

Fold set	Acc (%)	TPR (%)	TNR (%)	PPV (%)	MCC (%)
1	80.08	76.76	86.17	74.02	60.62
2	83.23	80.66	87.35	79.13	66.70
3	80.67	81.99	80.75	80.58	61.30
4	79.29	77.55	79.17	79.40	58.52
5	81.03	78.35	82.92	79.32	62.16
6	82.81	82.49	83.46	82.14	65.62
7	83.00	84.31	82.38	83.67	66.02
8	83.00	79.77	85.77	80.52	66.21
9	79.84	78.79	81.89	77.78	59.72
10	81.81	85.21	80.22	83.69	63.72
Average	81.48 ± 1.48	80.59 ± 2.86	83.01 ± 2.71	80.03 ± 2.88	63.06 ± 2.99

**Table 3:** The tenfold cross-validation details on Fdataset

Fold set	Acc (%)	TPR (%)	TNR (%)	PPV (%)	MCC (%)
1	78.04	75.94	78.02	78.05	56.00
2	79.84	84.86	75.85	84.44	60.20
3	79.59	82.56	78.16	81.22	59.25
4	78.04	75.46	83.59	72.40	56.37
5	77.26	78.37	79.13	75.14	54.30
6	82.17	80.39	84.97	79.38	64.45
7	77.20	73.17	81.97	72.91	54.91
8	76.17	76.00	77.55	74.74	52.32
9	76.94	73.33	79.44	74.76	54.08
10	73.06	71.20	73.51	72.64	46.11
Average	77.83 ± 2.43	77.13 ± 4.37	79.22 ± 3.48	76.57 ± 4.06	55.80 ± 4.93

As the Table 2 shown, the mean accuracy of tenfold cross-validation on Cdataset is 81.48% with standard deviation 1.48%, the mean TPR is 80.59% with standard deviation 2.86%, the mean TNR is 83.01% with standard deviation 2.71%, the average PPV is 80.03% with standard deviation 2.88% and the mean MCC of DDIPred on Cdataset is 63.06% with standard deviation 2.99%. The rigorous cross validation results provided that our model have obvious predictive ability for predicting the associations between drugs and diseases.

The tenfold cross-validation performance of DDIPred on Fdataset is shown in Table 3. The average accuracy on Fdataset is 77.83% with standard deviation 2.43%, and the average TPR is 77.13% with standard deviation 4.37%, the average TNR is 79.22% with standard deviation 3.48%, the average PPV is 76.57% with standard deviation 4.06% and the mean MCC of DDIPred on Fdataset is 55.80% with standard deviation 4.93%. The performance of DDIPred on this dataset is slightly weaker than on the Cdataset, but it still has acceptable results, which means it is competent for the drug–disease associations prediction task.

### Comparison with other state-of-the-art methods

**Fig. 3:**
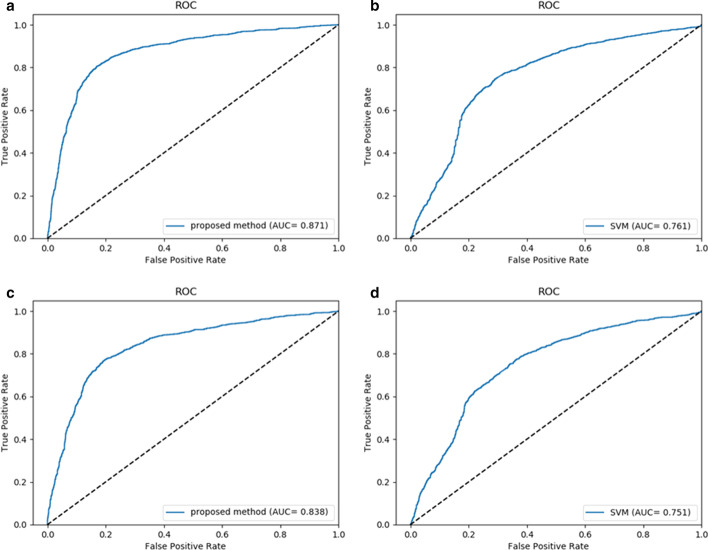
The performance of DDIPred and comparison method on two benchmark datasets: **a** the ROC and AUC of DDIPred on Cdataset; **b** the ROC and AUC of SVM on Cdataset; **c** the ROC and AUC of DDIPred on Fdataset; **d** the ROC and AUC of SVM on Fdataset

We further compared the proposed model with other state-of-the-art methods on same datasets under same experimental conditions, including previous studies and widely used machine learning model Support Vector Machine (SVM), the comparison results are reported at Tables 4 and 5 and Fig. 3.

**Table 4:** Comparison of the AUC of previous studies and DDIPred on datasets

Predictors	Cdataset	Fdataset
DrugNet	0.804	0.778
HGBI	0.858	0.829
DDIPred	**0.871**	**0.838**

Boldface indicates this measure of performance is the best among the compared methods

**Table 5:** Comparing the tenfold cross-validation performance of DDIPred and SVM on two gold standard datasets

Datasets	Methods	Acc (%)	TPR (%)	TNR (%)	PPV (%)	MCC (%)
Cdataset	SVM	72.57	70.99	76.41	68.70	45.25
	DDIPred	**81.48**	**80.59**	**83.01**	**80.03**	**63.06**
Fdataset	SVM	70.15	69.06	73.00	67.34	40.36
	DDIPred	**77.83**	**77.13**	**79.22**	**76.57**	**55.80**

Boldface indicates this measure of performance is the best among the compared methods

We compared the AUC of our model and previous studies including DrugNet [33] and HGBI [32]. Considering the difference of experimental evaluation indicators in different research, we only compared the AUC value reported in every study, which can best reflect the performance of model. As shown in Table 4 and Fig. 3, the DrugNet obtained a AUC of 0.804 on Cdataset and a AUC of 0.778 on Fdataset. The HGBI performed better than DrugNet with AUC of 0.858, 0.829 on Cdataset and Fdataset respectively. However, the AUC of DDIPred are 0.871, 0.838 on Cdataset and Fdataset, our model performs best on both datasets.

Furthermore, we did a comparison between our model and widely used machine learning model SVM, which is often used as a baseline model and usually has great performance in various fields. The feature input, tenfold cross validation set, evaluation metrics and other experimental conditions are exactly same between DDIPred and SVM model. The parameters of SVM are determined by grid search. The results are shown in Table 5. Our model has significantly improved all indicators.

### Case studies

In order to further examined the capability of the proposed model in predicting new associations between drugs and diseases. A drug and a disease are selected as case to be measured. The feature of the tested drug or disease and the feature of each disease or drug were combined as test data. Then, these data are fed into trained model to obtained prediction scores. Finally, all candidates are ranked based on prediction scores. The Zoledronic acid (DrugBank Accession Number: DB00399) and Dexamethasone (DrugBank Accession Number: DB01234) were selected for our case. Zoledronic acid is usually used to treat bone metastases pain, hypercalcemia of malignancy. And it can also helpful to prevent skeletal fractures in multiple myeloma and prostate cancer patients. Dexamethasone has anti-inflammatory, anti-immune, anti-toxin, antipyretic and other effects, and has a greater impact on metabolism. The prediction results are demonstrated in Tables 6 and 7, our model found the diseases most relevant to the target drugs, both confirmed indications and new potential candidate diseases are successfully predicted.

**Table 6:** Predicted diseases most relevant to Zoledronic acid

Rank	Indications	Disease ID
1	**MYELOMA, MULTIPLE**	**D254500**
2	**HAJDU-CHENEY SYNDROME**	**D102500**
3	**IBMPFD 1**	**D167320**
4	**HYPERCALCEMIA, INFANTILE**	**D143880**
5	**PAGET DISEASE OF BONE 2, EARLY-ONSET**	**D602080**
6	**MISMATCH REPAIR CANCER SYNDROME**	**D276300**
7	**HEREDITARY LEIOMYOMATOSIS AND RENAL CELL CANCER**	**D605839**
8	RENAL CELL CARCINOMA, NONPAPILLARY	D144700
9	OSTEOPOROSIS	D166710
10	ACROOSTEOLYSIS	D102400

Boldface indicates confirmed diseases, and normal font indicates the predicted candidate diseases

**Table 7:** Predicted diseases most relevant to Dexamethasone

Rank	Indications	Disease ID
1	**DERMATOSIS PAPULOSA NIGRA**	**D125600**
2	**MISMATCH REPAIR CANCER SYNDROME**	**D276300**
3	**ENTEROPATHY, FAMILIAL, WITH VILLOUS EDEMA AND IMMUNOGLOBULIN G2 DEFICIENCY**	**D600351**
4	**OTITIS MEDIA, SUSCEPTIBILITY TO**	**D166760**
5	**THROMBOCYTOPENIC PURPURA, AUTOIMMUNE**	**D188030**
6	**ASTHMA, NASAL POLYPS, AND ASPIRIN INTOLERANCE**	**D208550**
7	**MYCOSIS FUNGOIDES**	**D254400**
8	**DOHLE BODIES AND LEUKEMIA**	**D223350**
9	**HYPERTHERMIA, CUTANEOUS, WITH HEADACHES AND NAUSEA**	**D145590**
10	**GROWTH RETARDATION, SMALL AND PUFFY HANDS AND FEET, ANDECZEMA**	**D233810**
11	**GREENBERG DYSPLASIA**	**D215140**
12	ADIE PUPIL	D103100
13	ANEMIA, AUTOIMMUNE HEMOLYTIC	D205700
14	ATAXIA, EARLY-ONSET, WITH OCULOMOTOR APRAXIA AND HYPOALBUMINEMIA	D208920
15	ENDOMETRIOSIS, SUSCEPTIBILITY TO, 1	D131200

Boldface indicates confirmed diseases, and normal font indicates the predicted candidate diseases

## Conclusion

In this work, we proposed a novel deep learning model DDIPred using comprehensive similarity measure and Gaussian interaction profile kernel and gated recurrent neural networks to predict potential drug–disease associations, which may find new indications of existing drugs and can accelerate the process of drug research and development. The similarity measure matrix is used to exploit discriminative feature for drugs based on their chemical fingerprints. Meanwhile, the Gaussian interactions profile kernel is employed to obtain efficient feature for diseases based on known disease–disease associations. Then, we implemented a competitive deep learning GRU model to deal with the prediction task. Our model achieved remarkable performance on both two benchmark datasets with excellent AUC of 0.871 and 0.838 on Cdataset and Fdataset, and outperforms all comparison state-of-the-art models in many indicators. And we further made case studies to verify the predictive ability of our model. The rigorous experimental results proved the proposed method is powerful tool for predicting new indications for drugs or new treatments for diseases, and can be regarded as a useful guide for drug repositioning and drug discovery.

## Data Availability

The datasets used and/or analysed during the current study are available at: https://github.com/haichengyi/DDIPred.

## Abbreviations

R&D: Research and development
GIP: Gaussian interaction profile kernel
TSVD: Truncated Singular Value Decomposition
GRU: Gated Recurrent Units
SVM: Support Vector Machine
OMIM: Online Mendelian Inheritance in Man
SMILES: Simplified Molecular Input Line Entry Specification
LSTM: Long short-term memory
Acc: Accuracy
TPR: True positive rate
TNR: True negative rate
PPV: Positive predictive value
MCC: Matthews Correlation Coefficient
ROC: Receiver operating characteristic curve
AUC: The area under the receiver operating characteristic curve

## References

[1] Ashburn TT, Thor KB (2004). Drug repositioning: identifying and developing new uses for existing drugs. Nat Rev Drug Discov.

[2] Booth B, Zemmel R (2004). Prospects for productivity. Nat Rev Drug Discov.

[3] Dudley JT, Deshpande T, Butte AJ (2011). Exploiting drug–disease relationships for computational drug repositioning. Brief Bioinform.

[4] Nagaraj AB, Wang QQ, Joseph P, Zheng C, Chen Y, Kovalenko O, Singh S, Armstrong A, Resnick K, Zanotti K (2018). Using a novel computational drug-repositioning approach (DrugPredict) to rapidly identify potent drug candidates for cancer treatment. Oncogene.

[5] Luo H, Wang J, Li M, Luo J, Peng X, Wu FX, Pan Y (2016). Drug repositioning based on comprehensive similarity measures and Bi-Random walk algorithm. Bioinformatics.

[6] Luo H, Li M, Wang S, Liu Q, Li Y, Wang J (2018). Computational drug repositioning using low-rank matrix approximation and randomized algorithms. Bioinformatics.

[7] Chen X, Sun Y-Z, Zhang D-H, Li J-Q, Yan G-Y, An J-Y, You Z-H: NRDTD: a database for clinically or experimentally supported non-coding RNAs and drug targets associations. Database. 2017;2017:bax057. 10.1093/database/bax057 PMC5527270 29220444

[8] Kanehisa M, Goto S, Furumichi M, Tanabe M, Hirakawa M (2009). KEGG for representation and analysis of molecular networks involving diseases and drugs. Nucleic Acids Res.

[9] Hamosh A, Scott AF, Amberger J, Bocchini C, Valle D, Mckusick VA (2005). Online Mendelian Inheritance in Man (OMIM), a knowledgebase of human genes and genetic disorders. Nucleic Acids Res.

[10] Lamb J, Crawford ED, Peck D, Modell JW, Blat IC, Wrobel MJ, Lerner J, Brunet J-P, Subramanian A, Ross KN (2006). The Connectivity Map: using gene-expression signatures to connect small molecules, genes, and disease. Science.

[11] Knox C, Law V, Jewison T, Liu P, Ly S, Frolkis A, Pon A, Banco K, Mak C, Neveu V (2011). DrugBank 30: a comprehensive resource for ‘Omics’ research on drugs. Nucleic Acids Res.

[12] Kuhn M, Szklarczyk D, Pletscher-Frankild S, Blicher TH, Von MC, Jensen LJ, Bork P (2014). STITCH 4: integration of protein-chemical interactions with user data. Nucleic Acids Res.

[13] Gaulton A, Bellis LJ, Bento AP, Chambers J, Davies M, Hersey A, Light Y, Mcglinchey S, Michalovich D, Al-Lazikani B (2012). ChEMBL: a large-scale bioactivity database for drug discovery. Nucleic Acids Res.

[14] Meng F-R, You Z-H, Chen X, Zhou Y, An J-Y (2017). Prediction of drug–target interaction networks from the integration of protein sequences and drug chemical structures. Molecules.

[15] Luo H, Chen J, Shi L, Mikailov M, Zhu H, Wang K, He L, Yang L (2011). DRAR-CPI: a server for identifying drug repositioning potential and adverse drug reactions via the chemical–protein interactome. Nucleic Acids Res.

[16] Guo Z-H, You Z-H, Huang D-S, Yi H-C, Chen Z-H, Wang Y-B (2020). A learning based framework for diverse biomolecule relationship prediction in molecular association network. Commun Biol.

[17] Yi H-C, You Z-H, Huang D-S, Li X, Jiang T-H, Li L-P (2018). A deep learning framework for robust and accurate prediction of ncRNA-protein interactions using evolutionary information. Mol Ther Nucleic Acids.

[18] Yi H-C, You Z-H, Cheng L, Zhou X, Jiang T-H, Li X, Wang Y-B (2020). Learning distributed representations of RNA and protein sequences and its application for predicting lncRNA-protein interactions. Comput Struct Biotechnol J.

[19] He T, Bai L, Ong Y. Manifold regularized stochastic block model. In: 2019 IEEE 31st international conference on tools with artificial intelligence (ICTAI). 2019. P. 800–7.

[20] He T, Chan KCC (2018). Discovering fuzzy structural patterns for graph analytics. IEEE Trans Fuzzy Syst.

[21] He T, Chan KCC (2018). MISAGA: an algorithm for mining interesting subgraphs in attributed graphs. IEEE Trans Cybern.

[22] He T, Chan KCC (2019). Measuring boundedness for protein complex identification in PPI networks. IEEE/ACM Trans Comput Biol Bioinf.

[23] He T, Liu Y, Ko TH, Chan KCC, Ong YS. Contextual correlation preserving multiview featured graph clustering. IEEE Trans Cybern. 2020;50(10):4318–4331. 10.1109/TCYB.2019.2926431 31329151

[24] Yi H-C, You Z-H, Huang D-S, Guo Z-H, Chan KC, Li Y (2020). Learning representations to predict intermolecular interactions on large-scale heterogeneous molecular association network. iScience.

[25] Yi H-C, You Z-H, Guo Z-H (2019). Construction and analysis of molecular association network by combining behavior representation and node attributes. Front Genet.

[26] Chiang AP, Butte AJ (2009). Systematic evaluation of drug–disease relationships to identify leads for novel drug uses. Clin Pharmacol Ther.

[27] Gottlieb A, Stein GY, Ruppin E, Sharan R (2011). PREDICT: a method for inferring novel drug indications with application to personalized medicine. Mol Syst Biol.

[28] Francesco N, Yan Z, Moreira VM, Roberto T, Juha K, Mauro DA, Dario G (2013). Drug repositioning: a machine-learning approach through data integration. J Cheminform.

[29] Iorio F, Bosotti R, Scacheri E, Belcastro V, Mithbaokar P, Ferriero R, Murino L, Tagliaferri R, Brunetti-Pierri N, Isacchi A (2010). Discovery of drug mode of action and drug repositioning from transcriptional responses. Proc Natl Acad Sci.

[30] Cheng F, Liu C, Jiang J, Lu W, Li W, Liu G, Zhou W, Huang J, Tang Y (2012). Prediction of drug–target interactions and drug repositioning via network-based inference. PLoS Comput Biol.

[31] Wu C, Gudivada RC, Aronow BJ, Jegga AG (2013). Computational drug repositioning through heterogeneous network clustering. BMC Syst Biol.

[32] Wang W, Yang S, Zhang X, Li J (2014). Drug repositioning by integrating target information through a heterogeneous network model. Bioinformatics.

[33] Martínez V, Navarro C, Cano C, Fajardo W, Blanco A (2015). DrugNet: Network-based drug–disease prioritization by integrating heterogeneous data. Artif Intell Med.

[34] Zeng X, Zhu S, Liu X, Zhou Y, Nussinov R, Cheng F (2019). deepDR: a network-based deep learning approach to in silico drug repositioning. Bioinformatics.

[35] Chen H, Cheng F, Li J (2020). iDrug: Integration of drug repositioning and drug-target prediction via cross-network embedding. PLoS Comput Biol.

[36] Wishart DS, Knox C, Guo AC, Cheng D, Shrivastava S, Tzur D, Gautam B, Hassanali M (2008). DrugBank: a knowledgebase for drugs, drug actions and drug targets. Nucleic Acids Res.

[37] Steinbeck C, Han Y, Kuhn S, Horlacher O, Luttmann E, Willighagen E (2003). The Chemistry Development Kit (CDK): an open-source Java library for chemo-and bioinformatics. J Chem Inf Comput Sci.

[38] Weininger D (1988). SMILES, a chemical language and information system. 1. Introduction to methodology and encoding rules. J Chem Inf Comput Sci.

[39] Vanunu O, Magger O, Ruppin E, Shlomi T, Sharan R (2010). Associating genes and protein complexes with disease via network propagation. PLoS Comput Biol.

[40] Nepusz T, Yu H, Paccanaro A (2012). Detecting overlapping protein complexes in protein–protein interaction networks. Nat Methods.

[41] Yu L, Huang J, Ma Z, Zhang J, Zou Y, Gao L (2015). Inferring drug-disease associations based on known protein complexes. BMC Med Genomics.

[42] van Laarhoven T, Nabuurs SB, Marchiori E (2011). Gaussian interaction profile kernels for predicting drug–target interaction. Bioinformatics.

[43] Chen X, Jiang Z-C, Xie D, Huang D-S, Zhao Q, Yan G-Y, You Z-H (2017). A novel computational model based on super-disease and miRNA for potential miRNA–disease association prediction. Mol BioSyst.

[44] Hochreiter S, Schmidhuber J (1997). Long short-term memory. Neural Comput.

[45] Gers FA, Schmidhuber J, Cummins F. Learning to forget: continual prediction with LSTM. 1999. 10.1162/089976600300015015 11032042

[46] Shen Z, Bao W, Huang D-S (2018). Recurrent neural network for predicting transcription factor binding sites. Sci Rep.

[47] Yi H-C, You Z-H, Zhou X, Cheng L, Li X, Jiang T-H, Chen Z-H (2019). ACP-DL: a deep learning long short-term memory model to predict anticancer peptides using high-efficiency feature representation. Mol Ther Nucleic Acids.

[48] Wang Y-B, You Z-H, Yang S, Yi H-C, Chen Z-H, Zheng K (2020). A deep learning-based method for drug-target interaction prediction based on long short-term memory neural network. BMC Med Inform Decis Mak.

[49] Cho K, Van Merriënboer B, Bahdanau D, Bengio Y: On the properties of neural machine translation: Encoder-decoder approaches. arXiv preprint arXiv:14091259. 2014.

[50] Chung J, Gulcehre C, Cho K, Bengio Y: Empirical evaluation of gated recurrent neural networks on sequence modeling. arXiv preprint arXiv:14123555. 2014.

[51] Chollet F. Keras: The python deep learning library. Astrophysics Source Code Library. 2018.

[52] Gal Y, Hron J, Kendall A. Concrete dropout. 2017. arXiv preprint arXiv:1705.07832.

[53] Kingma DP, Ba J. Adam: a method for stochastic optimization. 2014. arXiv preprint arXiv:1412.6980v3.

[54] Yi H-C, You Z-H, Guo Z-H, Huang D-S, Chan KCC. Learning representation of molecules in association network for predicting intermolecular associations. IEEE/ACM Trans Comput Biol Bioinform. 2020. 10.1109/TCBB.2020.2973091. 10.1109/TCBB.2020.2973091 32070992

[55] Yi H-C, You Z-H, Wang M-N, Guo Z-H, Wang Y-B, Zhou J-R (2020). RPI-SE: a stacking ensemble learning framework for ncRNA-protein interactions prediction using sequence information. BMC Bioinform.

